# Gene Regulatory Networks Controlling Hematopoietic Progenitor Niche Cell Production and Differentiation in the *Drosophila* Lymph Gland

**DOI:** 10.1371/journal.pone.0041604

**Published:** 2012-07-24

**Authors:** Yumiko Tokusumi, Tsuyoshi Tokusumi, Douglas A. Shoue, Robert A. Schulz

**Affiliations:** Department of Biological Sciences, University of Notre Dame, Notre Dame, Indiana, United States of America; University of Massachusetts Medical School, United States of America

## Abstract

Hematopoiesis occurs in two phases in *Drosophila*, with the first completed during embryogenesis and the second accomplished during larval development. The lymph gland serves as the venue for the final hematopoietic program, with this larval tissue well-studied as to its cellular organization and genetic regulation. While the medullary zone contains stem-like hematopoietic progenitors, the posterior signaling center (PSC) functions as a niche microenvironment essential for controlling the decision between progenitor maintenance versus cellular differentiation. In this report, we utilize a PSC-specific GAL4 driver and UAS-gene RNAi strains, to selectively knockdown individual gene functions in PSC cells. We assessed the effect of abrogating the function of 820 genes as to their requirement for niche cell production and differentiation. 100 genes were shown to be essential for normal niche development, with various loci placed into sub-groups based on the functions of their encoded protein products and known genetic interactions. For members of three of these groups, we characterized loss- and gain-of-function phenotypes. Gene function knockdown of members of the BAP chromatin-remodeling complex resulted in niche cells that do not express the *hedgehog* (*hh*) gene and fail to differentiate filopodia believed important for Hh signaling from the niche to progenitors. Abrogating gene function of various members of the insulin-like growth factor and TOR signaling pathways resulted in anomalous PSC cell production, leading to a defective niche organization. Further analysis of the *Pten*, *TSC1*, and *TSC2* tumor suppressor genes demonstrated their loss-of-function condition resulted in severely altered blood cell homeostasis, including the abundant production of lamellocytes, specialized hemocytes involved in innate immune responses. Together, this cell-specific RNAi knockdown survey and mutant phenotype analyses identified multiple genes and their regulatory networks required for the normal organization and function of the hematopoietic progenitor niche within the lymph gland.

## Introduction

Stem cells are a unique class of cells that possess the ability to maintain a quiescent state, self renew, or undergo commitment to defined differentiation programs [1]–[3]. Their importance to a developing or aging animal includes their ability to give rise to multiple differentiated cell types that generate, maintain, and/or repair diverse tissues within the organism. Stem cells are known to be responsive to both intrinsic and extrinsic signals that regulate their functional properties. As for a source of extrinsic cues, the stem cell niche has been defined as a local tissue microenvironment that provides instructive signals for the maintenance and regulation of stem cell populations. Through intensive studies in this field, numerous tissue-specific stem cell niches have been identified in genetic organisms such as *C. elegans*, *D. melanogaster*, and *M. musculus* [1]–[5]. These findings have facilitated an in depth investigation into stem and support cell functions in germ line, nervous system, intestinal, muscle, hair, and hematopoietic development within these species.

A stem cell-like hematopoietic progenitor niche has been identified in the lymph glands of *Drosophila* larvae [6], [7]. As to lymph gland organization, the medullary zone is populated by multi-potent blood cell precursors that express components of the Hedgehog (Hh) and JAK/STAT signaling pathways [6], [7], the Suppressor of Hairless [Su(H)] and U-shaped (Ush) transcriptional regulators [8], [9], and the Bag of Marbles (Bam) translational regulator [10]. These prohemocytes appear to be derived from stem cells activated during late embryogenesis or early larval development [11]. Upon inducement to enter a lineage-specific differentiation program, progenitors locate to a more lateral position as differentiation-primed intermediate progenitors [12] that express the pro-differentiation factor Yan [10], [13]. Completion of specific blood cell differentiation programs results in the production of mature plasmatocytes or crystal cells that populate the cortical zone [14], [15].

A final lymph gland domain is the posterior signaling center (PSC), a region composed of 30–40 cells that fail to generate mature hemocytes [15]. Cells of the PSC are specified due to the function of the homeotic protein Antennapedia (Antp) and maintained due to the activity of the Collier (Col) transcription factor [7], [16]. Recently, the Wingless (Wg), Decapentaplegic (Dpp), and InR/TOR signaling pathways have been shown to play a role in determining the exact number of cells present within the PSC [17], [18], [19]. These cells differentiate in two discernible ways: by expressing the Hh and Serrate signaling molecules [7], [20] and by forming numerous filopodia that extend amongst the progenitor population within the medullary zone [6], [7]. Gene expression and function analyses have demonstrated that PSC-specific Hh expression is positively regulated by the GATA factor Serpent (Srp) and negatively attenuated by the Su(H) and Ush transcriptional regulators, with Srp function also required for PSC cell elaboration of filapodial extensions [8].

Several studies have supported the belief that the PSC serves as an instructive niche for neighboring hematopoietic progenitors, controlling their decision as to maintaining a pluri-potent state versus initiating a hemocyte differentiation program. That is, Hh produced by the PSC acts in a non cell-autonomous way to maintain the progenitor population while preventing blood cell differentiation [7]. In lymph glands lacking the PSC or failing to express Hh, the precursor population is lost due to the premature differentiation of hemocytes [7], [8]. An Adenosine deaminase growth factor arising from differentiating cells of the cortical zone is also required for prohemocyte quiescence, functioning with the niche-derived Hh signal in maintaining the progenitor state [21]. Disruption of JAK/STAT pathway signaling due the absence of a PSC in *col* mutant lymph glands similarly results in the depletion of prohemocytes from the medullary zone, coupled with the abundant production of differentiated blood cells [6]. Furthermore, a recent study demonstrated that wasp infestation of *Drosophila* larvae induces oxidative stress in PSC cells, resulting in the secretion of the Spitz cytokine signal from the niche that triggers the differentiation of lamellocytes within the lymph gland as part of an innate immune response to the pathogen [22]. Together, these findings demonstrate the essential role of the PSC as an instructive niche that controls blood cell homeostasis within the lymph gland hematopoietic organ.

The study of the organization of this *Drosophila* hematopoietic progenitor niche, and the intercellular signaling therein, has revealed several parallels to the structure and function of the mammalian hematopoietic stem cell (HSC) niche [2], [23]. This observation prompted us to undertake a loss-of-function survey to identify additional genes required for proper niche formation and function. A PSC-specific GAL4 driver line was used to direct the expression of a collection of UAS-RNAi transgenes, so as to knockdown the function of individual genes in PSC cells. Due to the existence of a repertoire of specialized markers for niche cells, hematopoietic progenitors, and differentiated hemocytes [6]–[8], [10], we could readily assess the effect of abrogating the function of targeted genes in terms of niche cell production and differentiation, as well as the status of blood cell homeostasis within the lymph gland. In this report, we present the results of systematically knocking-down the function of 820 *Drosophila* genes in PSC cells. A total of 100 genes were shown to be required for normal niche development and/or differentiation, with various loci placed into sub-groups based on their encoding transcriptional regulators, chromatin remodeling factors, cell cycle regulators, translational regulators, or signal transduction pathway components.

## Results

### Design and Summary of Findings of an RNAi-based PSC-specific Gene Function Knockdown Analysis of 820 *Drosophila* Genes

**Figure 1:**
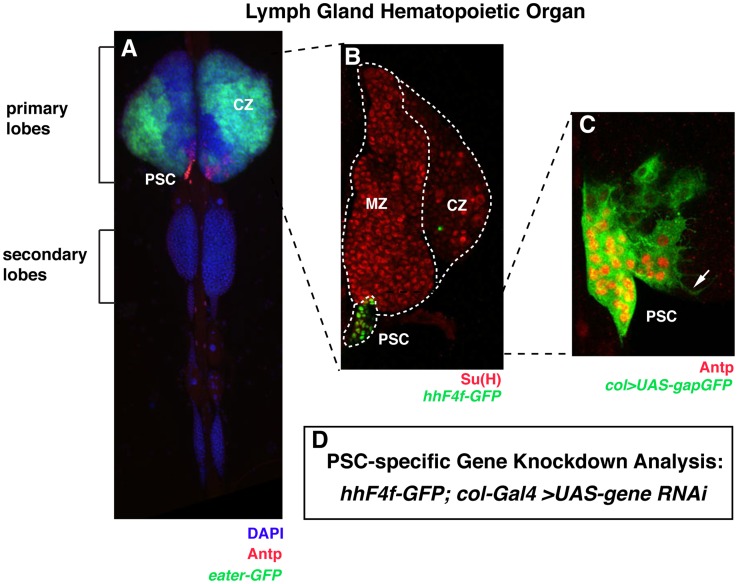
Lymph gland domains, cell markers, and strategy for a PSC-specific gene function knockdown analysis. (A) Dissected dorsal vessel and associated lymph glands assayed for DAPI (DNA), Antp protein (PSC cells), and *eater-GFP* transgene activity (plasmatocytes). Abbreviations: CZ, cortical zone; PSC, posterior signaling center. (B) Enlargement of a primary lymph gland lobe stained for Su(H) protein expressed in hematopoietic progenitors of the medullary zone (MZ) and crystal cells of the cortical zone (CZ). Also highlighted are *hhF4f-GFP*-positive niche cells of the PSC. (C) Focus on a lymph gland PSC niche assayed for nuclear Antp protein and membrane-associated GFP due to expression of the *col>UAS-gapGFP* combination. The arrow points out a filopodia extending from a niche cell. (D) Strategy for the PSC-specific gene function knockdown analysis undertaken in this study.

Blood cell production during the larval stage of development occurs in the lymph gland hematopoietic organ (Figure 1). Under normal growth and non-challenge conditions, the lymph gland maintains populations of hematopoietic progenitors in the medullary zone, differentiated hemocytes in the cortical zone, and niche-functioning cells within the posterior signaling center (PSC). Extensive studies on larval hematopoiesis has led to the generation of multiple high-resolution transgene and antibody markers for these distinct cell types, with the *hhF4f-GFP* transgene being a marker exclusive for the 30–40 niche cells of the PSC (Figure 1B) [8]. Such studies have also culminated in the generation of GAL4-driver strains that are cell-specific in their expression, including the PSC-precise *col-GAL4* line that utilizes the niche-active enhancer of the *collier* gene (Figure 1C) [6], [8], [16], [18]. Coupling *col-GAL4* activity with the wealth of RNAi transgenic lines available from *Drosophila* stock centers, a PSC-specific gene function knockdown analysis was initiated wherein we drove the expression of hairpin RNA that is processed by Dicer enzyme into siRNAs, so as to cause sequence-specific degradation of individual target gene mRNAs in niche cells (Figure 1D). *hhF4f-GFP* expression was the first marker assessed in the PSC cell-specific gene function knockdown analysis. As secondary markers, we assessed the status of Antp protein expression in PSC cell nuclei (Figure 1A, 1C) and the differentiation state of niche cells based on the presence or absence of extended filopodia, as monitored by *col>UAS-gapGFP*-directed membrane GFP expression (Figure 1C).

**Figure 2:**
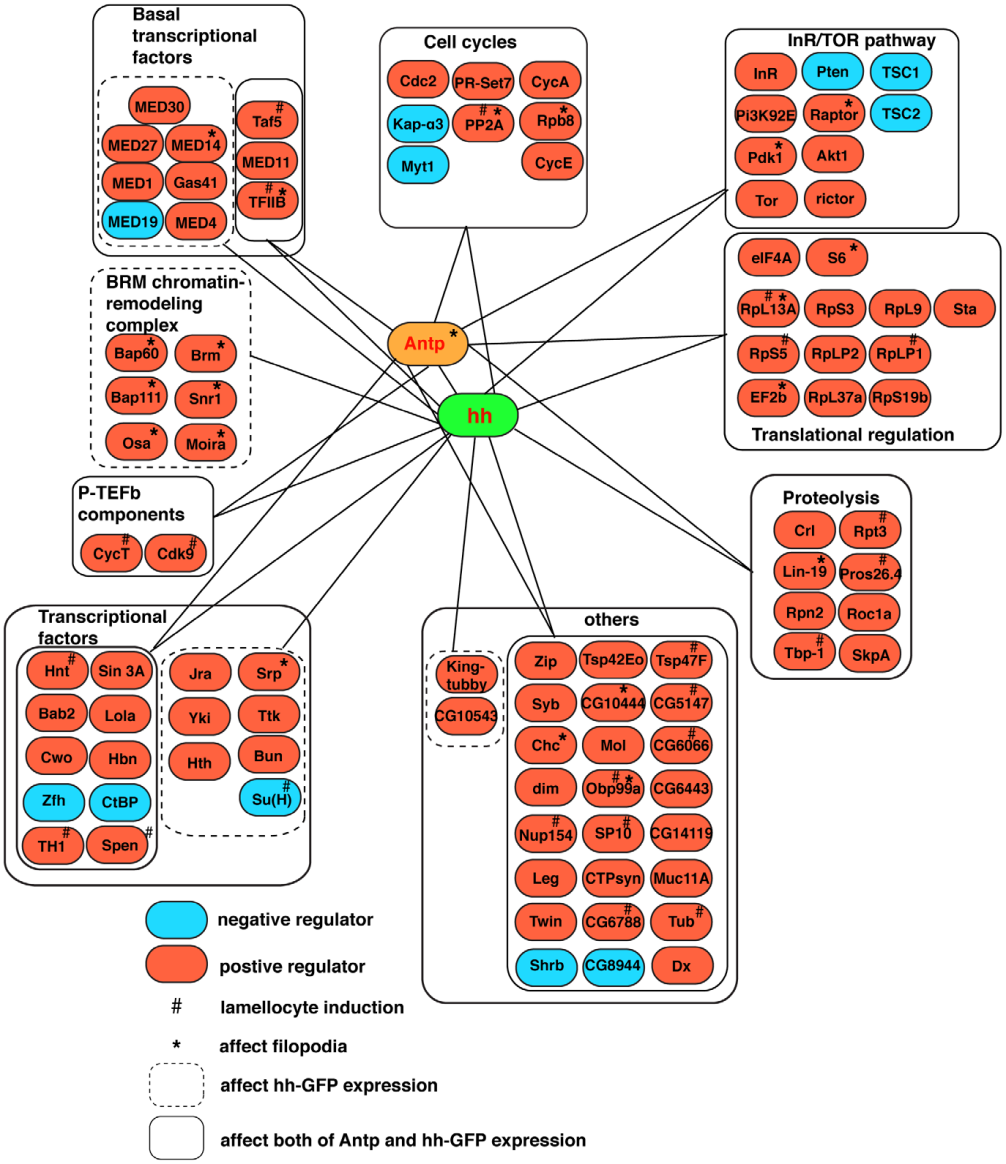
Summary of findings of a PSC-specific gene function knockdown analysis of 820 *Drosophila* loci. Center: *hhF4f-GFP* transgene activity (hh in green circle) was the primary marker assessed in the RNAi-based gene function knockdown analysis, while expression of Antp protein (Antp in yellow circle) and filopodia formation based on *col>UAS-gapGFP* expression were monitored as secondary markers. Periphery: Grouping of genes (based on their encoded protein products), that when functionally altered by RNAi expression in PSC cells, resulted in abnormalities in *hh-GFP*, Antp, and/or *col>UAS-gapGFP* expression. Negative (blue circle) and positive (red circle) regulators are indicated. A negative regulator is defined as a gene whose loss-of-function condition leads to increased numbers of *hhF4f-GFP*-positive cells and/or enhanced transgene expression, while a positive regulator is defined as a gene whose loss-of-function condition leads to decreased numbers of *hhF4f-GFP*-positive cells and/or decreased transgene expression. Those loss-of-function conditions that resulted in lamellocyte induction (#) or absence of filopodia (*) are also indicated.

A total of 820 *UAS-gene RNAi* transgenes were expressed in PSC cells (Table S1), with these transgenes chosen because of the known lymph gland expression of the individual genes based on microarray mRNA expression profiling [10]. Discernible differences in the PSC cell population were detected in the lymph glands of 100 of these *col>UAS-gene RNAi* larval cohorts (Figure 2, Table S2). Such abnormalities included an increased or decreased population of *hhF4f-GFP*-expressing cells, an increased or decreased population of Antp-expressing cells, scattered and disorganized niche cells, rounded cells lacking extended filopodia, and lamellocyte induction in the absence of a normal PSC. The 100 PSC-functioning genes were placed into sub-groups based on the function of their encoded protein products and known genetic interactions. These sub-groups included transcription factors, chromatin-remodeling complex components, cell cycle regulators, translational regulators, protein turnover regulators, and members of several distinct signal transduction pathways. We proceeded to analyze in detail the function of three of these gene sub-groups as to their requirements in the normal production, organization, and function of the hematopoietic progenitor niche.

### Requirement of BAP Chromatin-remodeling Complex Genes for Proper *hh* PSC Expression and Niche Cell Differentiation

**Figure 3:**
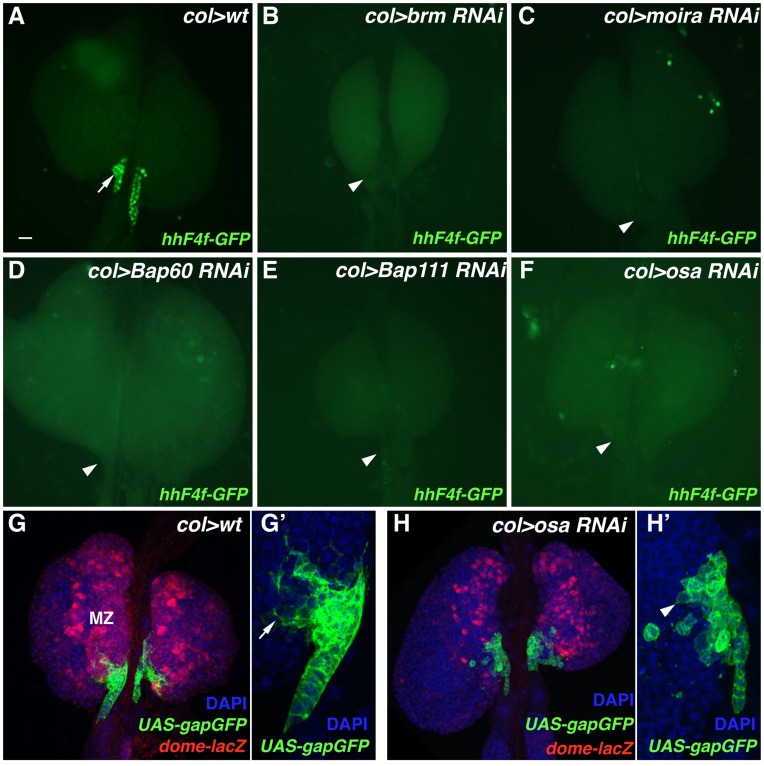
Gene function knockdown of BAP chromatin-remodeling complex genes. (A) *hhF4f-GFP* transgene activity in a wild-type lymph gland. Arrow points out GFP-positive PSC cells. (B–F) Lack of *hhF4f-GFP* transgene activity in lymph glands expressing *brm*, *moira*, *Bap60*, *Bap111*, or *osa* RNAi constructs, respectively. Arrowheads point out GFP-negative PSC cells in these tissues. (G) Wild-type lymph gland assayed for DAPI (DNA), *gapGFP* expression (differentiated niche cells), and *dome-lacZ* expression (hematopoietic progenitors of the medullary zone, MZ). (G’) Wild-type niche cells show normal differentiation as monitored by extension of filopodia (arrow). (H) PSC-specific RNAi knockdown of *osa* function results in a decreased population of *dome-lacZ*-positive hematopoietic progenitors and reduced *gapGFP*-positive PSC cells. (H’) *osa*-knockdown niche cells fail to differentiate filopodial processes (arrowhead). Scale bar indicates 20 µm.

SWI/SNF-related ATP-dependent chromatin remodeling complexes are known to control the accessibility of DNA sequences to transcription factors, thus facilitating the enhancement or repression of specific gene expression [24]. A distinct type of SWI/SNF-related complex in *Drosophila* is the BAP complex, which is defined by the presence of Osa, a DNA-binding protein of the Trithorax group [25]. Other subunits of this complex include the DNA-dependent ATPase Brahma (Brm), the Brm-associated proteins Bap60 and Bap111, the Snf5-related protein1 (Snr1), and Moira (Mor), which connects Brm and Snr1 within the complex. RNAi-based function knockdown of the genes encoding these six protein components resulted in an identical PSC phenotype, that being the lack of *hhF4f-GFP* transgene expression in niche cells (Figure 3B–3F). Based on Antp expression and *col>UAS-gapGFP* activity, the cells of the PSC are specified (Figure 3H) but fail to differentiate properly based on the absence of extended filopodia (Figure 3H’). Additionally, these mutant lymph glands contain reduced numbers of hematopoietic progenitors in the medullary zone (Figure 3H) and expanded populations of differentiated hemocytes (data not shown).

**Figure 4:**
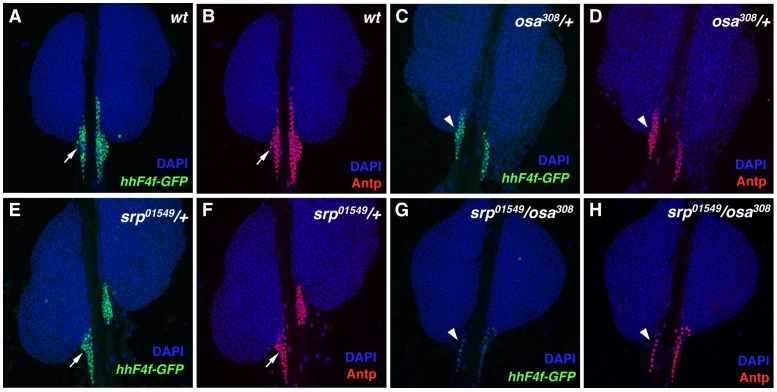
Genetic interaction between the *osa* and *srp* genes. (A) *hhF4f-GFP* transgene activity in PSC cells (arrow) of a wild-type lymph gland. (B) Antp expression in PSC cells (arrow) of a wild-type lymph gland. (C) *osa^308^/+* heterozygous lymph glands contain a decreased number of *hhF4f-GFP*-positive PSC cells (arrowhead). (D) *osa^308^/+* heterozygous lymph glands contain a decreased number of Antp-positive PSC cells (arrowhead). (E) Normal *hhF4f-GFP* transgene activity in PSC cells (arrow) of a *srp^01549^/+* heterozygous lymph gland. (F) Normal Antp expression in PSC cells (arrow) of a *srp^01549^/+* heterozygous lymph gland. (G) Absence of *hhF4f-GFP*-positive PSC cells (arrowhead) in a *srp^01549^/osa^308^* double-heterozygous lymph gland. (H) *srp^01549^/osa^308^* double-heterozygous lymph glands contain a reduced population of Antp-positive PSC cells (arrowhead).

Srp is a proven positive regulator of *hh* expression in niche cells [8]. Thus it was intriguing that the knockdown of BAP complex component functions in the PSC led to phenotypes also observed with the PSC-specific ablation of *srp* function: lack of *hh-GFP* expression, failure of niche cells to differentiate, and altered lymph gland blood cell homeostasis. Accordingly, we tested the possibility that Srp and Osa could functionally-interact in the regulation of niche cell *hh* expression. While wild-type (Figure 4A, 4B) and *srp^01549^/+* heterozygous (Figure 4E, 4F) lymph glands contain a normal population of *hhF4f-GFP*- and Antp-expressing PSC cells, *osa^308^/+* heterozygous (Figure 4C, 4D) tissues show a slightly reduced number of these cells. However, in *srp^01549^/osa^308^* double-heterozygous lymph glands, a greatly diminished population of Antp-positive niche cells is observed (Figure 4H), all of which fail to express the *hhF4f-GFP* transgene (Figure 4G). Such findings implicate the co-function of Srp and the BAP chromatin-remodeling complex in the positive regulation of Hh expression in the PSC, and possibly in the maintenance and differentiation of niche cells as well.

### Alteration of Insulin-like Growth Factor Signaling Pathway Gene Functions Results in Changed PSC Cell Number, Abnormal Niche Organization, and Disrupted Blood Cell Homeostasis in Mutant Lymph Glands

**Figure 5:**
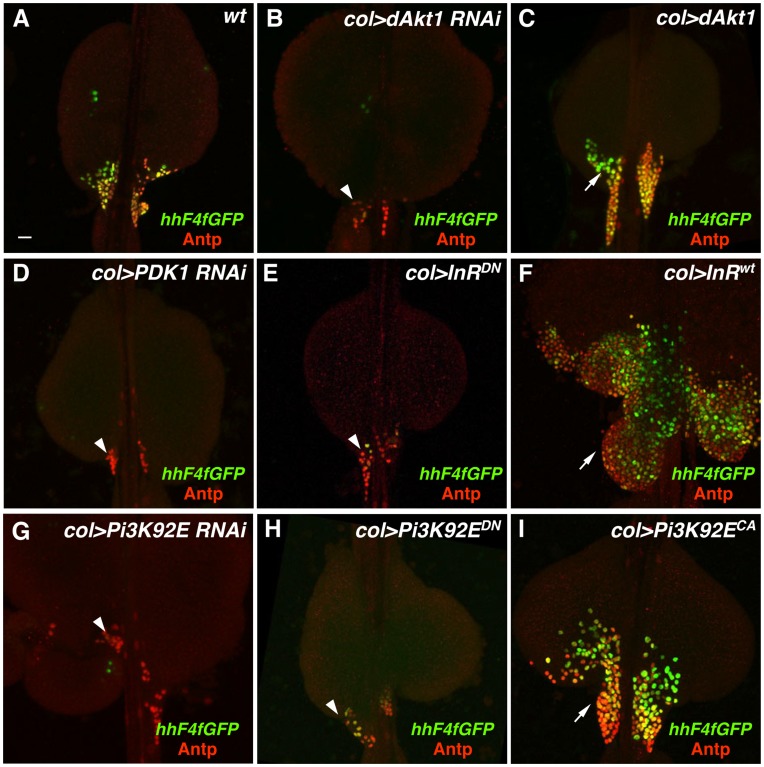
Alteration of insulin-like growth factor signaling pathway gene functions. Expression of the PSC cell-specific markers *hhF4f-GFP* and Antp was assessed in lymph glands of the following loss- or gain-of-function genotypes: (A) wild-type, (B) *col>dAkt1* RNAi, (C) *col>dAkt1* cDNA, (D) *col>PDK1* RNAi, (E) *col>InR^DN^* cDNA, (F) *col>InR* cDNA, (G) *col>Pi3K92E* RNAi, (H) *col>Pi3K92E^DN^* cDNA, and (I) *col>Pi3K92E^CA^* cDNA. Arrows in panels C, F, and I point out expanded populations of PSC niche cells, with an abnormal niche organization caused by PSC cell-specific expression of the *InR* cDNA. Arrowheads in panels B, D, E, G, and H point out reduced populations of PSC niche cells. Scale bar indicates 20 µm.

**Figure 6:**
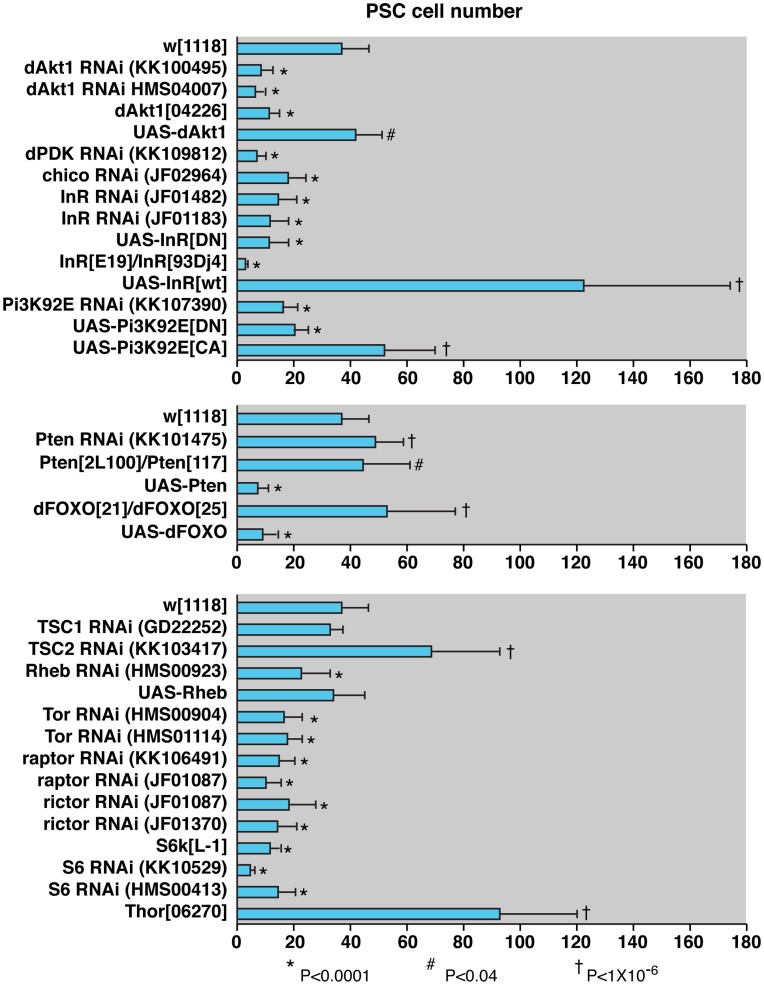
Quantification of PSC cell number.

An early finding of the PSC-specific RNAi analysis was the observation that lymph glands of the genotype *col>UAS-Akt1 RNAi* showed a severe depletion of *hh-GFP*- and Antp-positive cells (Figure 5B). This result was corroborated by a gain-of-function experiment as lymph glands obtained from *col>UAS-Akt1* animals showed a substantial increase in the number of *hh-GFP*- and Antp-positive PSC cells (Figure 5C). As the Akt1 protein kinase is a positive regulator within of the insulin-like growth factor signaling pathway, a pathway known to regulate organ growth and cell size [26], we tested the effect of altering the functions of other regulators of this growth control network. Genes that work positively in the insulin-like growth factor signaling pathway upstream of *Akt1* include *InR* that encodes the insulin receptor, *chico* that encodes an insulin receptor substrate, *Pi3K* that encodes the type 1A phosphatidylinositol 3-kinase, and *Pdk1* that encodes the Phosphoinositide-dependent kinase 1. RNAi knockdown of *Pdk1* (Figure 5D) or *Pi3k93E* (Figure 5G) function in the PSC resulted in depleted numbers of niche cells. Expression of dominant-negative forms of InR (Figure 5E) or Pi3K93E (Figure 5H) likewise resulted in a reduced PSC. The latter two findings were corroborated by the massive overgrowth and distortion of the niche in response to the forced expression of wild-type InR (Figure 5F) or constitutively-active Pi3K93E (Figure 5I). In total, we analyzed the consequence of 13 gain- or loss-of-function genotypes for these five positive regulators of the insulin-like growth factor signaling pathway, while quantifying the number of PSC niche cells (Figure 6). One conclusion from these studies is that genetic perturbations that positively regulate the signaling pathway and Akt1 activity result in supernumerary niche cell production, while genetic perturbations that negatively regulate the pathway and Akt1 activity result in a severe loss of PSC niche cells.

**Figure 7:**
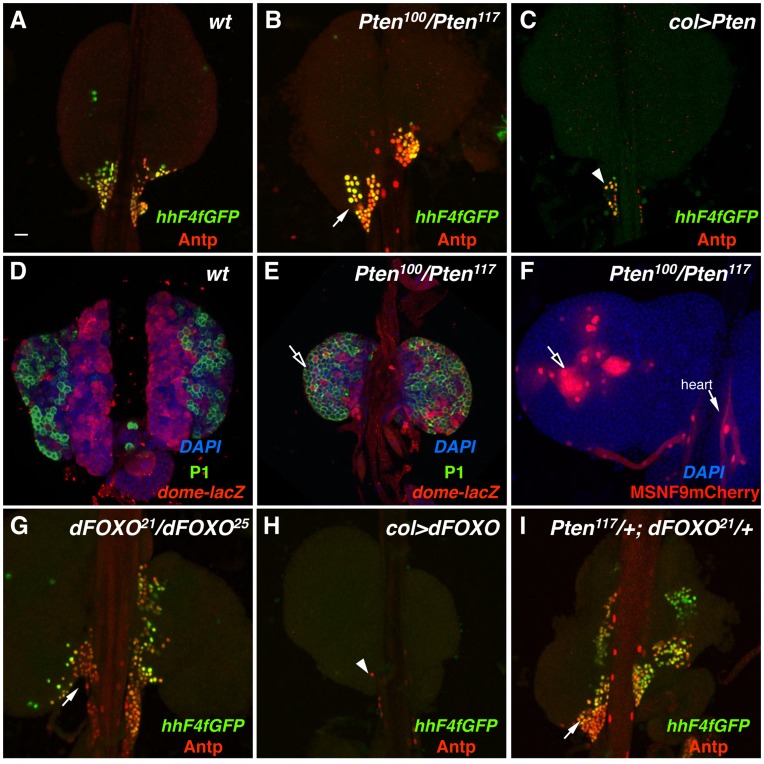
Alteration of *Pten* and *dFOXO* gene functions. Expression of the PSC cell-specific markers *hhF4f-GFP* and Antp was assessed in lymph glands of the following loss- or gain-of-function genotypes: (A) wild-type, (B) *Pten^100^/Pten^117^*, (C) *P85col>Pten* cDNA, (G) *dFOXO^21^/dFOXO^25^*, (H) *col>dFOXO* cDNA, and (I) *Pten^117^/+;dFOXO^21^/+*. Arrows in panels B, G, and I point out expanded populations and abnormal organization of niche cells. Arrowheads in panels C and H point out severely reduced populations of PSC niche cells. (D) Wild-type lymph gland assayed for DAPI (DNA), P1 antigen (plasmatocytes), and *dome-lacZ* expression (hematopoietic progenitors). (E) *Pten^100^/Pten^117^* lymph gland assayed for DAPI, P1 antigen, and *dome-lacZ* expression. The arrow points out the substantial increase in plasmatocyte number. (F) *Pten^100^/Pten^117^* lymph gland assayed for *MSNF9mCherry* transgene activity (lamellocytes). The arrow points out the de novo production of lamellocytes. Scale bar indicates 20 µm.

The *Pten* gene encodes a lipid phosphatase that negatively regulates the insulin-like growth factor signaling pathway through its inhibition of Pi3K activity. Thus it was not surprising that in the absence of *Pten* function in lymph glands, an expansion of PSC niche cell number was observed (Figure 7B), similar to the result obtained with the expression of *Pi3K92E^CA^* (Figure 5I). In contrast, the forced expression of *Pten* in PSC cells culminated in a substantial loss of the niche (Figure 7C). As a follow-up, we monitored the status of blood cell homeostasis in lymph glands mutant for *Pten* in all cells. Surprisingly, what was observed was a lack of maintenance of the hematopoietic progenitor population and the increase in the plasmatocyte population (Figure 7E) and induction of lamellocytes (Figure 7F). Thus the mutation of this tumor suppressor gene severely altered blood cell homeostasis, including the abundant production of lamellocytes, a specialized type of hemocyte involved in innate immune responses. This *Pten* hematopoietic phenotype is reminiscent of the mammalian knockout phenotype wherein hematopoietic stem cells are depleted and leukemia induced in a mouse *Pten* cancer model [27], [28]. Since this decrease of blood cell precursors occurred in *Pten* mutant lymph glands even though niche size is increased with the PSC-specific functional knockdown of *Pten*, we surmise that normal *Pten* function in hematopoietic progenitors is essential for their maintenance state.

Genetically downstream of these six genes is the FOXO transcription factor, which acts to positively regulate growth suppressors and negatively regulate translation-promoting genes in its control of organ growth and cell size [26]. One function for the Akt1 protein kinase is to phosphorylate FOXO, which leads to the inhibition of its transcriptional activator function. We observed that *FOXO* mutant lymph glands presented with a severe overgrowth and disorganization of the PSC niche (Figure 7G), while expression of FOXO in PSC cells resulted in a near complete loss of the niche (Figure 7H). Additionally, lymph glands that are double-heterozygous for *Pten* and *FOXO* alleles show an overgrowth and disorganization of the PSC (Figure 7I). These studies allowed us to conclude that the insulin-like growth factor signaling pathway plays a critical role in controlling niche cell number and organization, as well as hematopoietic progenitor maintenance versus blood cell differentiation during larval hematopoiesis.

### Alteration of TOR Signaling Pathway Gene Functions Results in Changed PSC Cell Number, Abnormal Niche Organization, and Disrupted Blood Cell Homeostasis in Mutant Lymph Glands

**Figure 8:**
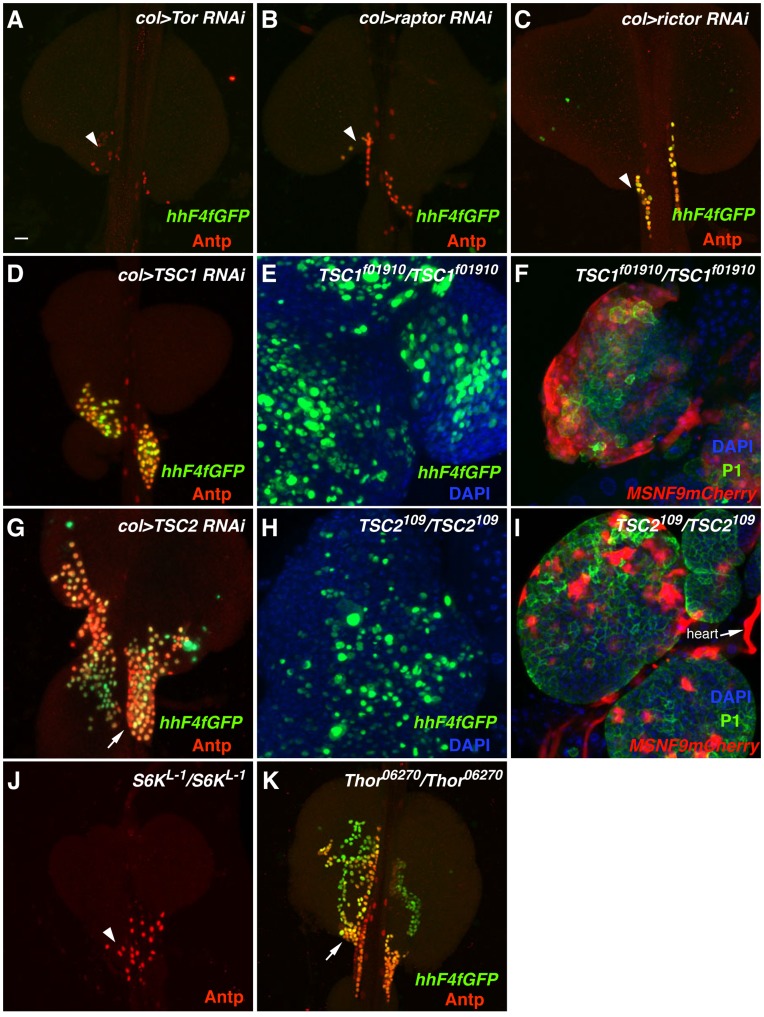
Alteration of TOR signaling pathway gene functions. Expression of the PSC cell-specific markers *hhF4f-GFP* and Antp was assessed in lymph glands of the following loss-of-function genotypes: (A) *col>Tor* RNAi, (B) *col>raptor* RNAi, (C) *col>rictor* RNAi, (D) *col>TSC1* RNAi, (G) *col>TSC2* RNAi, (J) *S6K^L-1^/S6K^L-1^* (Antp only), and (K) *Thor^06270^/Thor^06270^*. Arrows in panels G and K point out expanded populations and abnormal organization of PSC niche cells. Arrowheads in panels A, B, C, and J point out severely reduced populations of PSC niche cells. (E) Expanded expression of the *hhF4f-GFP* transgene in *TSC1^f01910^/TSC1^f01910^* lymph glands. (F) Increase in plasmatocyte number (detected by P1 antibody) and supernumerary lamellocyte production (detected by *MSNF9mCherry* activity) in *TSC1^f01910^/TSC1^f01910^* lymph glands. (H) Expanded expression of the *hhF4f-GFP* transgene in *TSC2^109^/TSC2^109^* lymph glands. (I) Increase in plasmatocyte number and supernumerary lamellocyte production in *TSC2^109^/TSC2^109^* lymph glands. Scale bar indicates 20 µm.

A major growth-regulatory target of insulin-like growth factor signaling is the Target of rapamycin (TOR) protein kinase, whose activity promotes ribosome biogenesis, translational initiation, and nutrient sensing [26]. Consistent with this activation function, knockdown of *Tor* function by PSC-specific RNAi expression results in a significant depletion of niche cells (Figure 8A). TOR functions in two distinct complexes, with the Raptor protein being a component of TORC1 and the Rictor protein being a component of TORC2 [29], [30]. RNAi knockdown of these two gene functions results in decreased numbers of PSC cells (Figure 8B, 8C), suggesting that both TOR complexes function to control PSC growth. TOR is directly activated by the small GTPase RHEB and PSC-specific RNAi knockdown of *Rheb* function likewise leads a diminished niche cell population (Figure 6).

**Figure 9:**
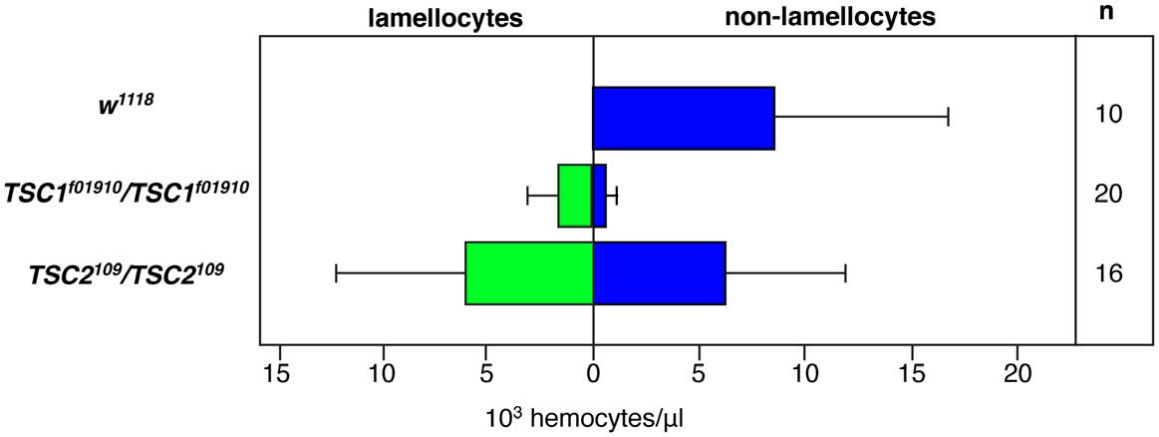
Quantification of hemolymph lamellocyte numbers.

*Drosophila* homologs of the Tuberous sclerosis complex (TSC) tumor suppressor genes *TSC1* and *TSC2* occupy a position upstream of TOR in this pathway that regulates cellular growth and metabolism [25]. The TSC1/TSC2 complex is negatively regulated by Akt1 function and this complex in turn negatively attenuates RHEB function in its activation of TOR. While PSC-restricted knockdown of *TSC1* gene function failed to cause a change in niche cell number (Figure 8D), knockdown of *TSC2* function did, leading to an overgrowth of the niche domain (Figure 8G). The further analysis of lymph glands that were mutant for viable *TSC1* or *TSC2* allelic combinations showed a pronounced de-repression of *hhF4GFP* transgene expression throughout the lymph glands (Figure 8E, 8H) and the extensive differentiation of plasmatocytes and lamellocytes in the mutant hematopoietic tissues (Figure 8F, 8I). We quantified lamellocyte numbers in wild-type versus *TSC1* or *TSC2* mutant third-instar larvae and observed these specialized immune cells composed about one-half of the circulating blood cell population within the hemolymph (Figure 9). As with the mutation of *Drosophila Pten*, the mutation of these two fly tumor suppressor genes culminates in the reduction of the hematopoietic progenitor population and the copious production of differentiated blood cells. This *TSC* hematopoietic phenotype is comparable to that observed with the targeted mutation of mouse *TSC1*, which disrupts HSC quiescence and their long-term function in blood cell production [31], [32].

Next we investigated three translational regulatory components that reside genetically downstream of the both the insulin-like growth factor and TSC-TOR signaling pathways. The S6K protein kinase is a common substrate of PDK1 and TORC1, where it activates ribosomal protein S6 and enhances protein synthesis. We observed that lymph glands of the genotype *S6K^L-1^/S6K^L-1^* and *col>S6 RNAi* presented with decreased numbers of niche cells (Figure 6, Figure 8J). *Thor* encodes the 4EBP translational and metabolic repressor protein, a gene activated by FOXO transcriptional regulation and a protein inhibited by the TOR protein kinase phosphorylation. Lymph glands with loss-of-function *Thor* showed a strong over-production of PSC cells that were present in an enlarged and extended niche domain (Figure 8K).

### Effect of Dietary Conditions on Lymph Gland Size and PSC Cell Number

**Figure 10:**
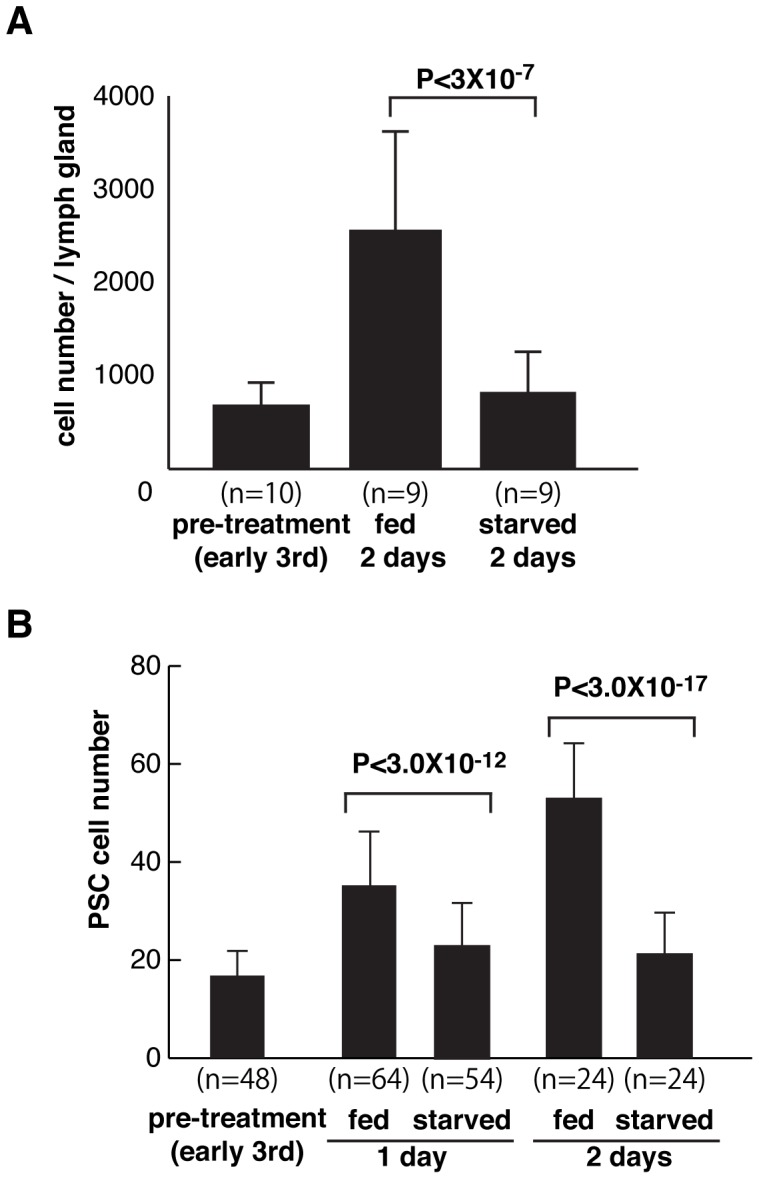
Quantification of lymph gland size and PSC cell number in fed or starved larvae. (A) Lymph gland cell number. (B) PSC cell number.

The insulin-like growth factor and TOR signaling pathways are known to coordinate nutrition with cell growth and animal size [25]. In our analysis of PSC cell number in insulin-like growth factor pathway mutants, we observed that *InR* and *Akt1* mutant larvae possessed lymph glands of reduced size (data not shown). Thus lymph gland growth is regulated by the activity of this signaling network. To determine if dietary conditions also affected lymph gland size and PSC cell number, we monitored tissue and niche size in wild-type control versus food-starved animals. As compared to normally fed larvae, lymph glands obtained from starved larvae were smaller in size (Figure 10A) and contained fewer PSC cells (Figure 10B). Thus it is a reasonable conclusion that the growth of the lymph gland hematopoietic organ, including the PSC domain, is controlled by nutrient sensing through the activities of the insulin-like growth factor and TOR signaling pathways. This finding was in contrast to a recent report that concluded larval starvation did not obviously alter the size of the lymph gland [33].

## Discussion

The discovery of a stem cell-like hematopoietic progenitor niche in *Drosophila* represents a significant contribution of this model organism to the study of stem cell biology and blood cell development. Extensive findings support the belief that the PSC functions as the niche within the larval lymph gland, with this cellular domain essential to the control of blood cell homeostasis within this hematopoietic organ [6]–[8], [22]. Molecular communication between the PSC and prohemocytes present in the lymph gland medullary zone is crucial for controlling the decision as to maintaining a pluri-potent progenitor state versus initiating a hemocyte differentiation program. This lymph gland cellular organization and the signaling pathways controlling hematopoieis therein have prompted several researchers in the field to point out its functional similarity to the HSC niche present in mammalian organisms [2], [23].

As a means to discover new information on genetic and molecular mechanisms at work within a hematopoietic progenitor niche microenvironment, we carried out an RNAi-based loss-of-function analysis to selectively eliminate individual gene functions in PSC cells. We assessed the effect of knocking-down the function of 820 lymph gland-expressed genes as to their requirement for niche cell production and differentiation, and 100 of these genes were shown to be required for one or more aspects of niche development. The distinguishable phenotypes observed in these analyses included change in number of Hh-expressing cells, change in number of Antp-expressing cells, scattered and disorganized niche cells, rounded cells lacking extended filopodia, and lamellocyte induction in the absence of a normal PSC. Figure 2 and Table S2 serve as compilations of the phenotype(s) induced with the functional knockdown of the 100 PSC-required genes. These genes were placed into sub-groups based on their coding capacity and known genetic interactions, and we characterized in greater detail the phenotypes associated with the functional knockdown of members of three of these gene regulatory networks.

We previously demonstrated that the PSC-specific ablation of *srp* function resulted in a lack of expression of the crucial Hh signaling molecule in these cells, the inactivity of the *hh-GFP* transgene in the niche, failure of niche cells to properly differentiate filopodial extensions, and the loss of hematopoietic progenitor maintenance coupled with the abundant production of differentiated hemocytes [8]. Thus we were intrigued when we observed that RNAi function knockdown of several members of the BAP chromatin-remodeling complex resulted in the identical phenotypes of lack of *hh-GFP* transgene expression and absence of filopodia formation in PSC cells. A convincing functional interaction was observed between *srp* encoding the hematopoietic GATA factor and *osa* encoding the DNA-binding Trithorax group protein in the inability of niche cells to express *hh-GFP* in double-heterozygous mutant lymph glands. Thus one working model is that the BAP chromatin-remodeling complex establishes a chromatin environment around and within the *hh* gene that allows access of the Srp transcriptional activator to the PSC-specific enhancer, facilitating Hh expression in these cells. It will be of interest to determine if there exists a direct physical interaction between Osa and Srp in this positive regulation of *hh* niche transcription and if so, what are the functional domains of the proteins essential for this critical regulatory event in progenitor cell maintenance. It is also likely that these functional interactions are important for Srp’s transcriptional regulation of additional genes needed for the formation of niche cell filapodia.

In this study, we analyzed a total of 33 gain- or loss-of-function genetic conditions that enhanced or eliminated the function of various positive or negatively-acting components of the insulin-like growth factor and TOR signaling pathways. A conclusion to be drawn from these analyses is that genetic conditions that have an end effect of enhancing translation activity and protein synthesis result in supernumerary PSC cell numbers in disorganized niche domains, while conditions that promote growth suppression lead to substantially reduced populations of niche cells. The same conclusion was obtained from recent studies performed by Benmimoun and colleagues [19]. The Wg and Dpp signaling pathways have also been shown to be important for the formation of a PSC niche of normal size and function [17], [18], and it is possible that the insulin-like growth factor and TOR signaling networks regulate the translation of one or more members of the Wg and/or Dpp pathways. Our analyses have also shown that mutation of the *Pten*, *TSC1*, and *TSC2* tumor suppressor genes results in severely altered blood cell homeostasis in lymph glands and in circulation, including the prolific induction of lamellocytes. A recent report demonstrated that in response to larval wasp infestation, the PSC secretes the Spitz cytokine signal, which triggers an EGFR-mediated signal transduction cascade in the generation of dpERK-positive lamellocytes in circulation [22]. As dpERK activity is known to inhibit TSC2 function [34], inactivation of the TSC complex may be a downstream regulatory event leading to robust lamellocyte production in larvae in response to wasp immune challenge.

To summarize, an RNAi-based loss-of-function analysis has been undertaken to identify new genes and their signaling networks vital for normal PSC niche formation and function. While we have gained information on the requirements of three such networks for PSC development and blood cell homeostasis within the lymph gland, numerous other genes have been discovered that likewise play key roles in these hematopoietic events. Their characterization is warranted as well to further enhance our knowledge of genetic and molecular mechanisms at work within an accessible and easily-manipulated hematopoietic progenitor niche microenvironment.

## Materials and Methods

### *Drosophila* Strains and Culture

Fly lines were cultured at 18°C or 25°C on cornmeal-based fly food. Transgenic RNAi lines obtained from the Vienna Drosophila RNAi Stock Center and the Bloomington Stock Center are listed in Table S1. The following strains were used: *col-Gal4* [6](M. Crozatier); *hhF4f-GFP* [8]; *eater-GFP*, *MSNF9mo-mCherry* [35]; *Pten^2L100^*, *Pten^117^*, *dFoxo^21^*, *dFoxo^25^*, *UAS-dFOXO* [36], [37] (E. Hafen); *S6k^l-1^* [38] (G. Thomas); *dome-lacZ* (*dome*-MESO) [39] (N. Fossett); *srp^01549^*, *osa^308^*, *Akt1^04226^*, *InR^E19^*, *InR^93Dj4^*, *Thor^06270^*, *TSC1^f01910^*, *gig*/*TSC2^109^*, *UAS-Akt1*, *UAS-InR^wt^*, *UAS-InR^DN^*, *UAS-Pi3K92E^CA^*, *UAS-Pi4K92E^DN^*, *UAS-Rheb*, *UAS-gapGFP* (from the Bloomington Stock Center). For starvation experiments, early third-instar larvae were carefully collected with forceps, washed with PBS, and transferred to new fly food or filter papers soaked with water on plastic dishes. After 1 or 2 days at room temperature, larvae were dissected and analyzed with the indicated blood cell markers.

### Immunostaining of *Drosophila* Lymph Glands

Tissue immunostainings were performed as described in previous studies [8], [10]. The following primary antibodies were used: rabbit anti-Akt1 (1∶100; Cell Signaling 9272), mouse anti-Antp (1∶100; 4C3, Developmental Studies Hybridoma Bank), and mouse anti-plasmatocyte (P1) antibody (1∶100; I. Ando) [40]. As secondary antibodies, we used Alexa 488 or 555-conjugated anti-rabbit or mouse IgG antibodies (Invitrogen). Immunostained samples were analyzed with a Zeiss Axioplan fluorescence microscope or a Nikon AR-1 laser-scanning confocal microscope.

### Cell Counting

To determine the concentration of circulating hemocytes and lamellocytes, counting and statistical analyses were performed as previously described [41], [42]. For PSC cell counting, anti-Antp antibody-stained lymph glands were used.

## Supporting Information

Table S1 **List of RNAi lines tested in this study.**
(XLS)

Table S2 **Results of PSC-specific gene function knockdown screening.** OTE is Off-target and is any gene hit by fewer than 50% of a construct’s sense 19-mers, but at least one (Vienna Drosophila RNAi Center). s19 is defined by the following formula: s19 = ∑ ON-target matches/(∑ ON-target matches +∑ OFF-target matches) (Vienna Drosophila RNAi Center). Highlight corresponds to many off-target sites and these RNAi results are removed from Fig. 2. “↓” indicates an RNAi reduces marker-positive cell number. “↑” indicates an RNAi increases marker-positive cell number. “weak” means an RNAi reduces marker expression level without affecting cell number. Enhanced hhGFP means hhGFP-positive cell number is not changed or reduced, but GFP expression is enhanced.
(XLS)
