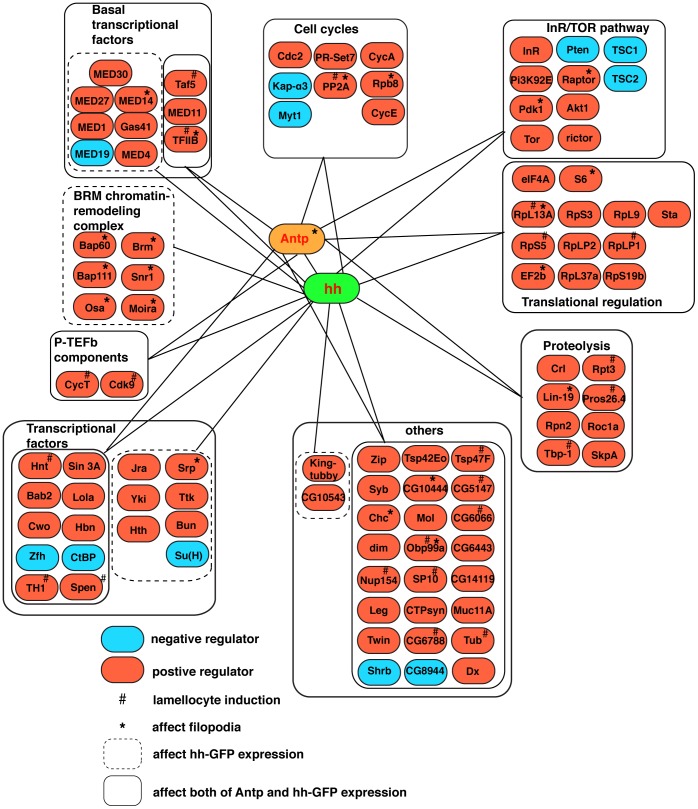# Correction: Gene Regulatory Networks Controlling Hematopoietic Progenitor Niche Cell Production and Differentiation in the *Drosophila* Lymph Gland

**DOI:** 10.1371/annotation/f9660803-198b-4d0d-8200-719a2eb2a443

**Published:** 2013-03-04

**Authors:** Yumiko Tokusumi, Tsuyoshi Tokusumi, Douglas A. Shoue, Robert A. Schulz

There was an error in Figure 2. The correct Figure 2 can be seen here: 

**Figure:**